# Oligosaccharide Presentation Modulates the Molecular Recognition of Glycolipids by Galectins on Membrane Surfaces

**DOI:** 10.3390/ph15020145

**Published:** 2022-01-26

**Authors:** Marta G. Lete, Antonio Franconetti, Sandra Delgado, Jesús Jiménez-Barbero, Ana Ardá

**Affiliations:** 1 CICbioGUNE, Basque Research & Technology Alliance (BRTA), Bizkaia Technology Park, Building 800, 48160 Derio, Bizkaia, Spain; mgutierrez@cicbiogune.es (M.G.L.); afranconetti@cicbiogune.es (A.F.); sdelgado@cicbiogune.es (S.D.); 2 Ikerbasque, Basque Foundation for Science, Maria Diaz de Haro 3, 48013 Bilbao, Bizkaia, Spain; 3 Department of Organic Chemistry, II Faculty of Science and Technology, University of the Basque Country, EHU-UPV, 48940 Leioa, Bizkaia, Spain; 4 Centro de Investigacion Biomedica En Red de Enfermedades Respiratorias, 28029 Madrid, Madrid, Spain

**Keywords:** lectins, presentation, NMR, molecular recognition, glycolipids, galectins

## Abstract

Galectins are a family of glycan binding proteins that stand out for the wide range of biological phenomena in which they are involved. Most galectin functions are associated with their glycan binding capacities, which are generally well characterized at the oligosaccharide level, but not at the glycoprotein or glycolipid level. Glycolipids form the part of cell membranes where they can act as galectin cellular receptors. In this scenario, glycan presentation as well as the membrane chemical and structural features are expected to have a strong impact in these molecular association processes. Herein, liposomes were used as membrane mimicking scaffolds for the presentation of glycosphingolipids (GSLs) and to investigate their interaction with Galectin-3 and the N-domain of Galectin-8 (Gal8N). The binding towards GM3 and GM1 and their non-silaylated GSLs was compared to the binding to the free glycans, devoid of lipid. The analysis was carried out using a combination of NMR methods, membrane perturbation studies, and molecular modeling. Our results showed a different tendency of the two galectins in their binding capacities towards the glycans, depending on whether they were free oligosaccharides or as part of GSL inserted into a lipid bilayer, highlighting the significance of GSL glycan presentation on membranes in lectin binding.

## 1. Introduction

Glycans are presented at cell surfaces attached to either proteins (glycoproteins) or lipids (glycolipids), where they can be recognized by carbohydrate-binding proteins (lectins) to mediate or influence diverse processes such as cell adhesion, signaling, intracellular trafficking, or pathogen recognition [1]. In mammals, the most common presentation of glycans on lipids occurs on a ceramide (Cer) scaffold to generate glycosphingolipids (GSLs). Ceramide structures vary in length, hydroxylation pattern, and degree of saturation of the fatty acid moieties, resulting in lipid structural diversity that impacts the presentation of the attached glycan at the membrane surface [2]. However, most structural diversity of glycolipids is provided by the glycan moiety. In vertebrates, the first sugar linked to Cer can be either Galactose (Gal), or Glucose (Glc), with the latter being typically glycosylated to give lactosylceramide (Galβ1-4GlcβCer, LacCer). This neutral core is the common structure for the ganglio-, lacto-, neolacto-, globo-, and isoglobo- GSL series, as found in mammals. Regardless of whether they are based on the ganglio-series sugar core, all GSLs containing at least one Neu5Ac residue are referred to as gangliosides [3].

Accumulating evidence indicates that many cellular events are highly influenced by gangliosides acting as cellular receptors [4]. For instance, GM1 or GD1a/b are known to be exploited by pathogens as ligands for bacterial toxins [5,6,7] and viral adhesins [8,9,10]. Most growth factor receptors are known to be regulated by gangliosides [11,12,13]. Tumor-associated gangliosides have been suggested to play important roles in malignant cancer progression, [4,14,15,16] and represent a potential opportunity to be exploited in cancer immunotherapy [17].

Lectins, in particular galectins and sialic acid-binding immunoglobulin-like lectins (Siglecs), [18] have been noted as functional protein receptors for gangliosides. It has been suggested that cell-surface enzymatic glycan remodeling modulate molecular recognition events and cellular function by shifting affinities between these two lectin families [18]. A compendium of work using mammalian cells have shown that galectins can bind GM1 present on the cell surface [19,20,21]. Other studies have correlated lectin, specifically Galectin-3 [22], binding to gangliosides with endocytic processes [23], leading to the so-called GlycoLipid-Lectin (GL-Lect) hypothesis, according to which, upon lectin-binding to glycolipids and glycoproteins, membrane curvature is induced, promoting the formation of endocytic pits, leading to clathrin-independent carriers (CLICs) [24,25,26].

Insights into the molecular basis of the interaction between galectins and GSLs has been provided by X-ray crystallography [27,28], although these studies have been carried out using only the glycan component, devoid of the lipid moiety. Moreover, most of our current knowledge about galectin-glycan binding preferences comes from surface-based assays [23,29] or affinity chromatography studies, [30] where glycan presentation is expected to strongly differ from that occurring for GSLs anchored at the cell membrane. In any case, these studies have shown that although galectins share the ability to recognize Gal moieties, the affinity and selectivity towards Gal-containing saccharides differ significantly, resulting in subtle but relevant diverse glycan binding preferences. Glycan presentation [31] and density [32], which have been shown to strongly affect the affinity and selectivity of these molecular recognition processes [33], are expected to become especially relevant for GSLs anchored at cell membranes where glycan epitope accessibility, dynamics, and conformational preferences are anticipated to dramatically differ from other glycan presentations [34,35], as those imposed by the above mentioned experimental settings. In spite of these evidences [18], there are very few detailed studies at high resolution on the interaction between lectins and gangliosides [36], probably due to the difficulties in handling glycolipids in a hydrophilic medium. In fact, our atomistic knowledge on these processes in the cellular context is still scarce. Recently, using self-assembled dendrimers, which form lamellar structures together with lipids, mixed vesicles were formed, named glycodendrimersomes, in order to study the distribution and their interaction with different galectins [37]. Regardless, this is still far from in vivo conditions.

The addition of Neu5Ac to lactose (α2-3 linked), as often found in the glycan moieties of gangliosides, has been shown to decrease the affinity for some galectins, while producing the opposite effect for others. Galectin-3 (Gal3) and the N-terminal domain of Gal-8 (Gal8N) are among the galectins that, in solution, show preference for the presence of Neu5Ac, being this effect much more pronounced for Gal8N [30]. Interestingly, Gal8N has been proposed to bind GM3 but not GM1 on cell surfaces [23], while Gal3 binding to GM1/GM3 containing liposomes was not observed unless it was chemically attracted to the lipid bilayer [22].

**Scheme 1:**
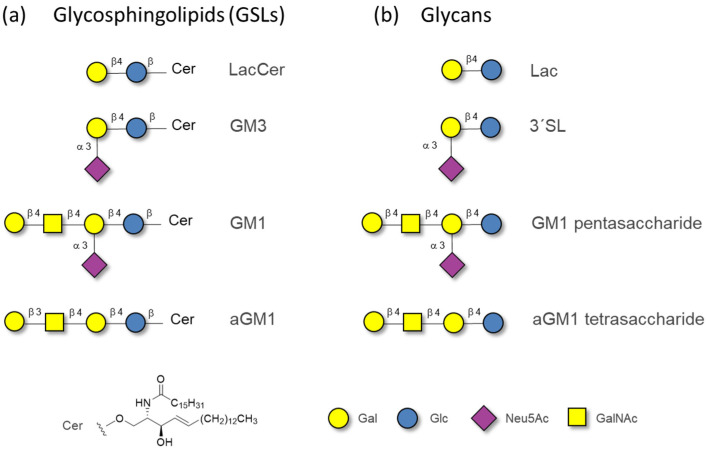
(**a**) GSLs and (**b**) glycan structures whose interaction with Gal3 and Gal8N are studied herein, along with the used nomenclature.

Herein, we have used liposomes as membrane-mimicking scaffolds for the presentation of GM1 and GM3 gangliosides, along with their corresponding non-sialylated GSLs, LacCer, and asialoGM1 (aGM1), for exploring their interaction features (Scheme 1a) with Gal3 and Gal8N. The binding was compared to that occurring in solution between the galectins and the isolated glycan moieties. Interestingly, different relative binding affinities were observed, depending on the presentation either as a free species or presented on the membrane. Therefore, we provide experimental evidence on how glycan presentation on cell membranes may impact their recognition features and eventually, their associated functions.

## 2. Results

### 2.1. The Binding of the Sugar Moieties to Galectin 3-and N-Terminal Domain of Galectin-8

**Figure 1:**
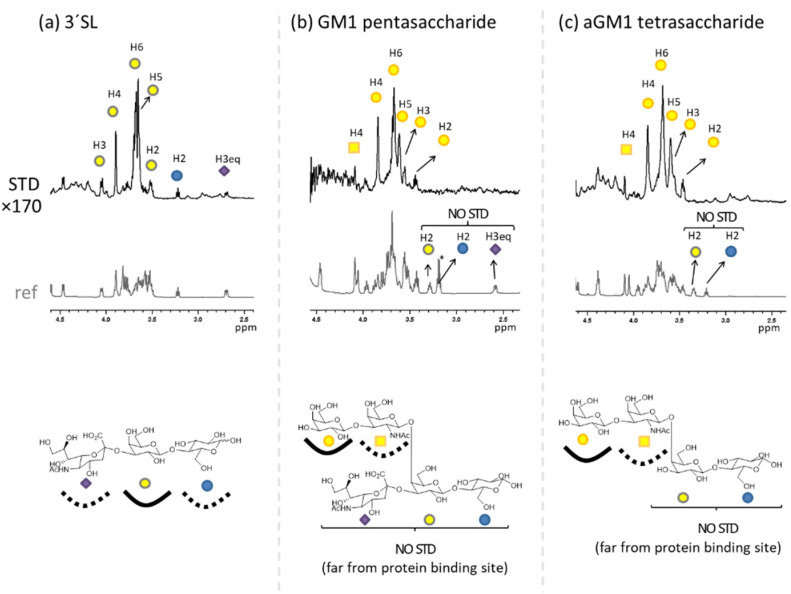
^1^H-STD-NMR and corresponding reference spectra for the interaction of glycans (**a**) 3′SL, (**b**) GM1 pentasaccharide, and (**c**) aGM1 tetrasaccharide with Gal-3 full length, and schematic representation of the STD NMR-based glycan binding epitopes (below): a solid curve indicates main binding epitope, and a dashed curve indicates additional minor contacts with the protein. Residues showing no STD NMR signals are far from the protein surface. The asterisk indicates and impurity (EtOH).

First, the binding in the solution of the saccharides (Scheme 1b) to Gal3 and Gal8N was studied by employing complementary NMR strategies. The glycan epitopes were deduced by employing ^1^H-STD-NMR experiments [38,39]. As expected [27], for 3’SL binding to Gal3, the most intense STD signals correspond to the Gal moiety, with weak signals for the Glc and Neu5Ac residues (Figure 1a). For the GM1 and aGM1 oligosaccharides, the most intense STD signals correspond to the terminal Gal residue, and no STD was observed for the internal Gal moiety, nor for Neu5Ac and Glc residues, clearly indicating that this part of the glycan is far from the protein’s surface, not contributing to the binding (Figure 1b,c). The fact that the STD spectrum of GM1 penta- and aGM1 tetra-saccharides are basically the same with respect to STD intensity and epitope mapping, further confirms that the Neu5Ac residue in GM1 do not contribute at all to the binding, in contrast to 3′SLN [40]. For the interaction with Gal8N, very similar results were obtained: again, the STD spectra for GM1 and aGM1 oligosaccharides were very similar, with no STD observed for the internal Gal and Glc residues in aGM1 and neither Neu5Ac in GM1 (Figure S1). Interestingly, the STD spectrum of Gal8N with 3′SL showed rather weak STD intensities, being the STD for H6-Gal and for H3-Neu5Ac almost no detectable (Figure S1b). We attribute this result to the higher affinity interaction Ga8N/3′SL (see below), which biased the STD effect for reporting on epitope mapping [39].

**Figure 2:**
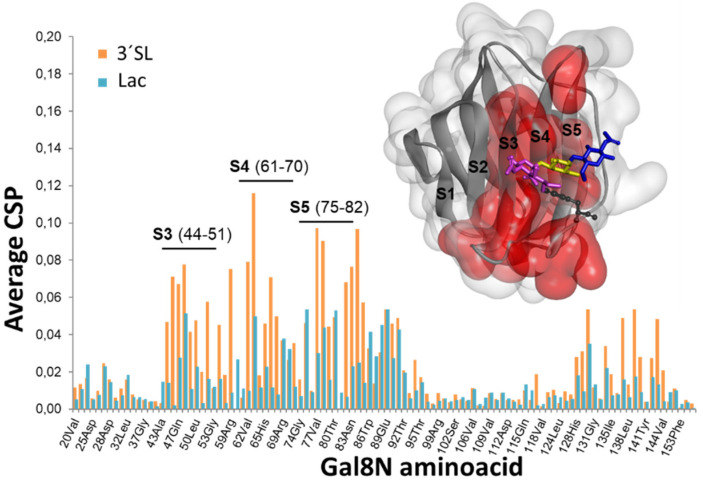
CSP plot measured for the interaction of Gal8N with lactose (in blue) and 3′SL (in orange) upon protein saturation for the 20 and 1.5 molar ratio complexes, respectively. The molecular model corresponds to the X-ray crystallographic structure of Gal8N/3′SLN (pdb 3vko), where the residues affected in the presence of 3′SL and not with lactose are highlighted in red. 3′SLN is represented as sticks with Neu5Ac in magenta, Gal in yellow, and GlcNAc in blue. The key W86 is represented in black as ball and sticks.

The lectins’ binding region as well as an estimation of the dissociation binding constants (K_D_) (Table 1) for the interaction of Gal3 and Gal8N with the saccharides (Scheme 1b) were then deduced by using standard ^1^H-^15^N-HSQC titration experiments, employing ^15^N labelled galectins. Lactose was also included in the analysis for comparative purposes. The observed chemical shift perturbation (CSP) of the backbone amide signals of Gal3 in the presence of either lactose or 3′SL are shown in Figure S2A. The presence of the Neu5NAc in 3′SL strongly affects a few localized residues at strand S5 and S4 and increased the binding affinity only slightly (by 1.8-fold) with respect to that of lactose (Table 1). On the contrary, the interaction with GM1 and aGM1 oligosaccharides (Figure S2B,C) produced similar CSP profiles and a slightly worsened (GM1 penta, by a 2.5-fold decreased aGM1 tetra), binding affinity with respect to lactose. For Gal8N, the binding to 3′SL was slow in the chemical shift timescale, while for the other three analogues it was in the fast exchange regime. In fact, the estimated affinity following intensity changes between the free and bound states [41] of Gal8N for 3′SL was ca. 30 stronger than for lactose, while for GM1 and aGM1 was in the same order of magnitude with respect to lactose. Moreover, the observed CSP pattern for Gal8N/3′SL (Figure 2) covered a much more extended region along the Gal8N sequence than that observed for Gal3, affecting almost all residues at strands S3, S4, and S5. When the interactions of the GM1 and aGM1 to Gal8N were compared (Figure S2E,F), the observed CSP (fast exchange) were again comparable to those for lactose. Overall, our data agree with X-ray crystallographic structures of Gal8N with 3′SLN (pdb 3vko) and Gal3 with 3′SL and GM1 (pdbs 4lbo and 3ayc), and with the reported binding affinity data [30,42,43]. It was shown that 3′SL was a better binder than GM1 and aGM1 sugars for both lectins, which displayed parallel affinity for these oligosaccharides to that for lactose, while Gal8N showed much increased affinity for 3′SL, with respect to lactose than Gal3 (30-fold and 1.8-fold, respectively).

### 2.2. The Binding of Galectins to Gangliosides in Model Membranes

In order to give a step forward towards a more natural scenario, the binding of Gal3 and Gal8N to GSL-containing liposomes was then analyzed. It was hypothesized that these GSL in liposomes somehow mimic the glycan presentation at the cell membrane. The liposomes were built by adding the different GSL, LacCer, GM3, GM1, or aGM1 to the lipid mixture (see Section 4) at either 1 or 10 equivalents, with respect to the lectin. Blank HSQC experiments were carried out using naked (with no GSL included) phosphatidylcholine (PC) liposomes.

First, a similar NMR spectroscopy approach, based on monitoring the changes in the ^1^H-^15^N HSQC spectra of the galectins in the absence and presence of the GSL-containing liposomes was attempted. However, no CSP were observed, but a significant reduction in the intensities of the lectin cross peaks, depending on the galectin and on the chemical nature of the binding partner. Although different effects can be behind this intensity decrease, we consider it highly likely that when the saccharide-lectin complex is formed, since the saccharide is attached to the liposome, the effective rotational correlation time of the system becomes very large, the transverse relaxation rate is fast, the signals broaden, and the cross-peak intensities decrease. In fact, the observed reduction in the signal intensities was a general trend for all the HSQC cross peaks, with no specificity for those corresponding to amino acid residues at the binding site. Thus, the data were analyzed by comparing the average peak volumes for all the lectin signals.

**Figure 3:**
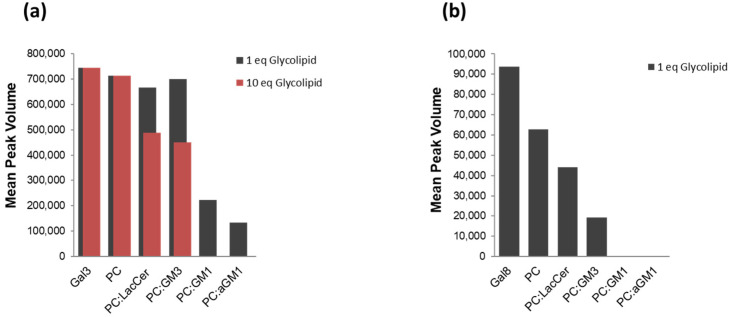
Quantification of the peak volume reduction of (**a**) ^15^N-Gal3 CRD and (**b**) ^15^N-Gal8N upon binding to liposomes containing only PC or the glycolipids LacCer, GM3, GM1, or aGM1. For Gal3, 1 eq and 10 eq of the corresponding glycolipid with respect to the protein is shown, while for Gal8N only the data with 1 eq glycolipid are shown, as with 10 equivalents all the HSQC cross peaks vanished.

For Gal3, no reduction in the cross-peak intensities was observed in the presence of the naked PC liposomes (Figure 3, Gal3 vs. PC bars), while a subtle, although measurable, reduction was detected when the liposomes contained one equivalent of LacCer or GM3 respect to Gal3 (Figure 3a, grey bars). Further decrease of the intensities were perceived when 10 equivalents of these gangliosides respect to Gal3 were present in the liposomes (Figure 3a, red bars), evidencing the existence of lectin binding to the sugar moieties on the liposomes. A different behavior was observed when the liposomes contained GM1 or aGM1. In these cases, the cross-peak volumes were already significantly reduced with just one equivalent of ganglioside (more than 50% in average), as shown in Figure 3a, and in the presence of the liposomes containing 10 equivalents of GM1 or aGM1, all the lectin cross-peaks completely vanished in the HSQC spectrum. This evidence suggests that, under these experimental conditions, Gal3 binds stronger to GM1 or aGM1 than to LacCer or GM3. Interestingly, this is the opposite trend to that observed for the free oligosaccharides (Table 1). Under the experimental conditions described herein, when this glycan is presented in the membrane mimetic, the highest affinities are shown by GM1/aGM1.

Strikingly, a different behavior was also observed for Gal8N. In this case, a noticeable reduction (ca. 35%) of the HSQC cross peak intensities was already observed for the blank experiment, in the presence of the naked liposomes (Figure 3b, Gal8 vs. PC bars). This nonspecific interaction is probably due to the negatively charged patch found in the opposite face to the binding site of the protein that could interact with the choline groups of the membrane surface. This patch is present in Gal8N, but not in hGal3-CRD, where that area of the protein is neutral (Figure S3 in Supplementary Materials). When only one equivalent of LacCer or GM3 respect to Gal8N was introduced in the liposomes, a further reduction in the cross-peak intensities was observed (Figure 3b). The presence of just one equivalent of GM1 or aGM1 erased all the backbone amide signals in the HSQC spectrum, leaving only a few cross peaks for some Asn/Gln side chains. In all cases, the presence of 10 equivalents of glycolipid respect to the lectin produced the disappearance of all the NMR signals. Therefore, these experiments suggested not only that the binding tendency for Gal8N was similar to that for Gal3- CRD, with a higher affinity for GM1 and aGM1 than for LacCer and GM3, but that this galectin domain also displayed some interactions with the membrane-like environment.

Competition experiments were then performed to deduce the selectivity of the binding event. Particularly, to a solution of the complex formed by the GSL-containing liposomes and the lectin (either Gal3 or Gal8N) at 1:1 molar ratio, different molar equivalents of a given competitor (a known glycan ligand) were added, and the HSQC spectrum recorded again for the mixture. For the complexes generated with LacCer, lactose was added; for the GM3-containing liposomes, 3′SL was added; for the GM1 and aGM1-containing liposomes, the disaccharide of the T-antigen epitope (Galβ1-3GalNAc) was added [44].

**Figure 4:**
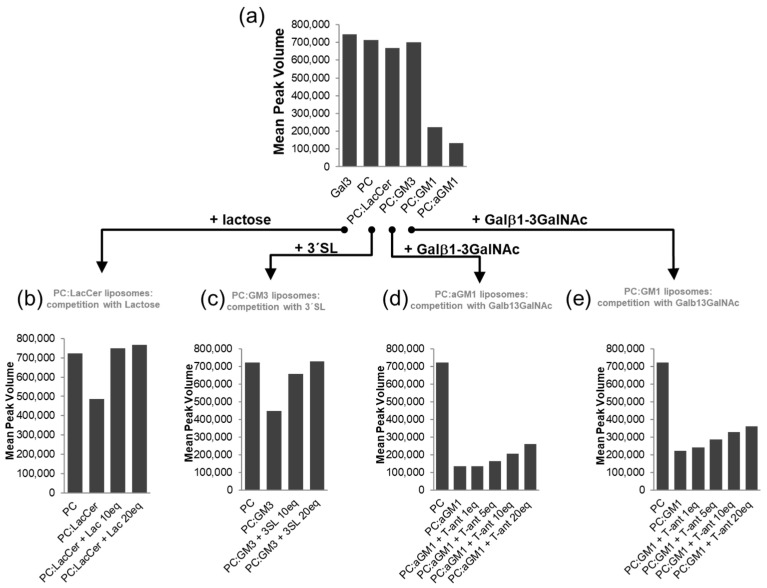
Competition experiments with Gal3-CRD. (**a**) Peak volume reduction of ^15^N-Gal3 CRD in the presence of differently functionalized liposomes: PC only or one equivalent (with respect to the lectin) of glycolipid (LacCer, GM3, GM1, or aGM1); (**b**) Peak volume recovery of ^15^N-Gal3 CRD/LacCer containing liposomes upon the addition of 10 and 20 equivalents of lactose as binding competitor; (**c**) Peak volume recovery of ^15^N-Gal3 CRD/GM3 containing liposomes upon the addition of 10 and 20 equivalents of 3′SL as binding competitor; (**d**) Peak volume recovery of ^15^N-Gal3 CRD/aGM1 containing liposomes upon the addition of 10 and 20 equivalents of Galβ1-3GalNAc as binding competitor; (**e**) Peak volume recovery of ^15^N-Gal3 CRD/GM1 containing liposomes upon the addition of 10 and 20 equivalents of Galβ1-3GalNAc as binding competitor.

For the Gal3/LacCer and Gal3/GM3 systems, the addition of 10 equivalents of lactose (Figure 4b) or 3′SL (Figure 4c) restored the initial intensities of the cross peaks volumes and provided the expected CSP observed in solution (HSQC spectra in the supporting information). Instead, when the external epitope of GM1/aGM1, Galβ1-3GalNAc (up to 20 equivalents) was added (Figure 4d,e) to the solutions containing either the Gal3/GM1 or the Gal3/aGM1 systems, the peak volume was not completely restored (up to 40–50% maximum recovery). Once more, in contrast to the observations in solution, this experimental evidence further supports the existence of better binding of Gal3 CRD for GM1 and aGM1 than for LacCer and GM3, when the interacting epitopes are presented as GSL on a lipid membrane.

**Figure 5:**
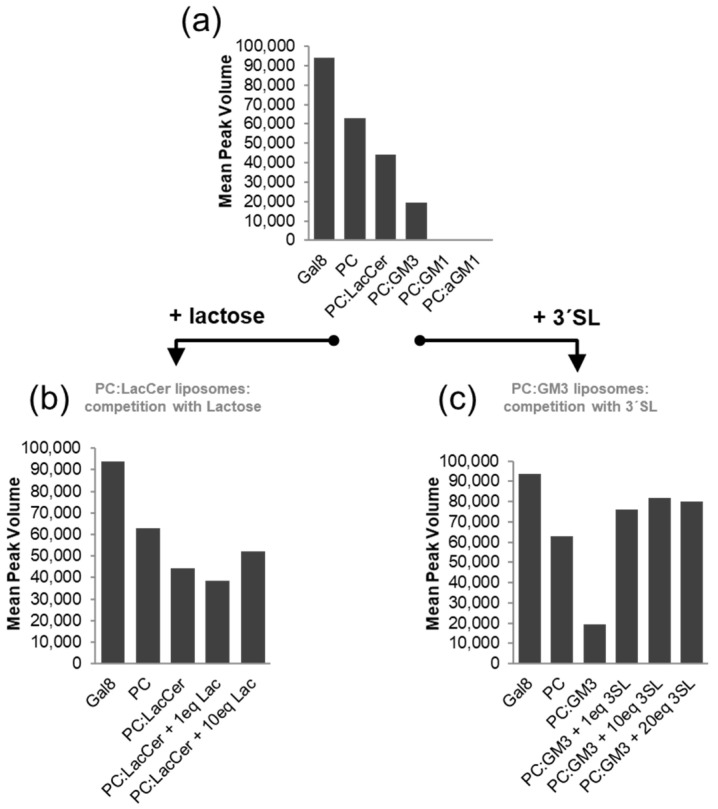
Competition experiments with Gal8-N. (**a**) Peak volume reduction of ^15^N-Gal8-N in the presence of differently functionalized liposomes: PC only, or one equivalent (with respect to the lectin) of glycolipid (LacCer, GM3, GM1, or aGM1). (**b**) Peak volume recovery of ^15^N-Gal8N/LacCer containing liposomes upon the addition of 10 and 20 equivalents of lactose as binding competitor; (**c**) Peak volume recovery of ^15^N-Gal8N/GM3 containing liposomes upon the addition of 10 and 20 equivalents of 3′SL as binding competitor.

For hGal8-Nter, a similar situation occurred for the LacCer-containing liposome. The addition of 10 equivalents of lactose to the NMR sample with Gal8N/ LacCer system only provided a partial recovery (ca. 80%) of the HSQC intensities (Figure 5A). In contrast, the addition of one equivalent of 3SL to the sample containing the mixture of the GM3-containing liposome (one eq) with the lectin provided the complete recovery of the initial cross-peak volumes.

### 2.3. Membrane Perturbation

**Figure 6:**
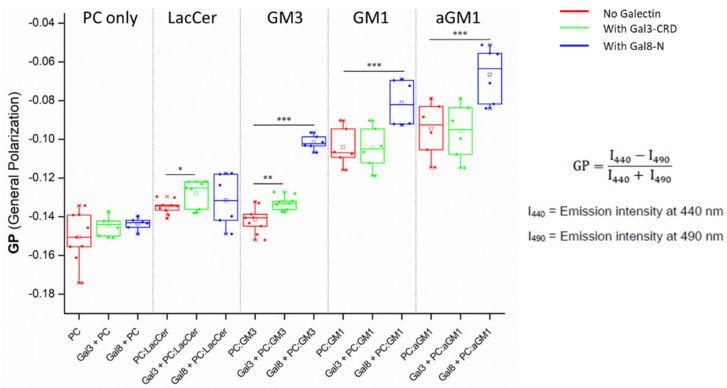
Laurdan GP values for membrane with/without GSLs upon the addition of Gal3 and Gal8N. Liposome content is indicated above. *** *p* < 0.001, ** *p* < 0.01, * *p* < 0.05 of unpaired *t* test.

In order to explore the possibility of membrane perturbation effects on the liposomes (naked and GSL-loaded) by the presence of the galectins, the fluorescent probe Laurdan was employed (Figure 6) [45]. This dye is extremely sensitive to solvent relaxation effects, and by following the shift in the emission spectrum, it can be used to monitor changes in membrane bending, phase transitions, hydration, domain formation, or protein-lipid interactions. Laurdan’s naphthalene moiety localizes at the level of the glycerol backbone of phospholipids. When excited by incident light, this functional group undergoes an important charge separation, creating a strong dipole moment. Dipolar relaxation with the surrounding water molecules at the level of phospholipid glycerol backbones seems to be the cause of the large emission maximum shift changes, which can be quantified by calculating the generalize polarization (GP). Laurdan in liquid phase (PC) membranes was introduced together with the gangliosides. Next, Gal3-CRD was added to the preparations. No significant changes in the GP values (Figure 6, red vs. green) were observed when liposomes contained GM1 or aGM1, which indicates that Gal3 directly binds to the terminal Gal moiety of these GSLs without perturbing the membrane (Scheme 1). In contrast, for LacCer- and GM3-containing liposomes, a change in the GP values was evidenced with statistical significance. These data strongly suggest that for LacCer and GM3, the carbohydrate moiety is near the membrane and the binding to Gal3 promotes a small perturbation of the surface.

Instead, when Gal8N was added a spectacular change in the GP value was observed for the GM1-, aGM1-, and especially GM3-containing liposomes (Figure 6 red vs. blue). For LacCer, no significant changes were noted. These highlight that the membrane surface is perturbed when Gal8N recognizes the terminal corresponding glycan binding epitopes in GM1, aGM1, and especially in GM3 (Scheme 1), but it is not altered by the presence of only Gal8N or when Gal8N binds to the LacCer-containing liposomes. While the NMR data (Figure 5) suggests some sort of non-glycan mediated interaction between Gal8N and the naked liposomes, the lack of GP changes indicates that this interaction does not significantly perturb the membrane surface, supporting the fact that it is a non-specific electrostatic interaction (see above).

All these facts indicate the existence of a complex interplay between the lectin, the glycan presentation, and the liposome, which is manifested in the experimental observations. In order to try to provide a structural explanation to these experimental observations, MD simulations were then carried out.

### 2.4. Molecular Simulation of Membranes Containing Glycolipids

Molecular Dynamics (MD) simulations were then performed to provide a 3D view of the presentation and dynamics of the four glycolipids (LacCer, GM3, aGM1, and GM1) embedded in a dynamic POPC bilayer. The employed protocol for the preparation of the system is described in the Section 4. Briefly, five different systems were built: system 1 contained only a POPC bilayer, while systems 2, 3, 4, and 5 contained a POPC lipid bilayer with 16 inserted molecules of LacCer, GM3, aGM1, and GM1, respectively.

**Figure 7:**
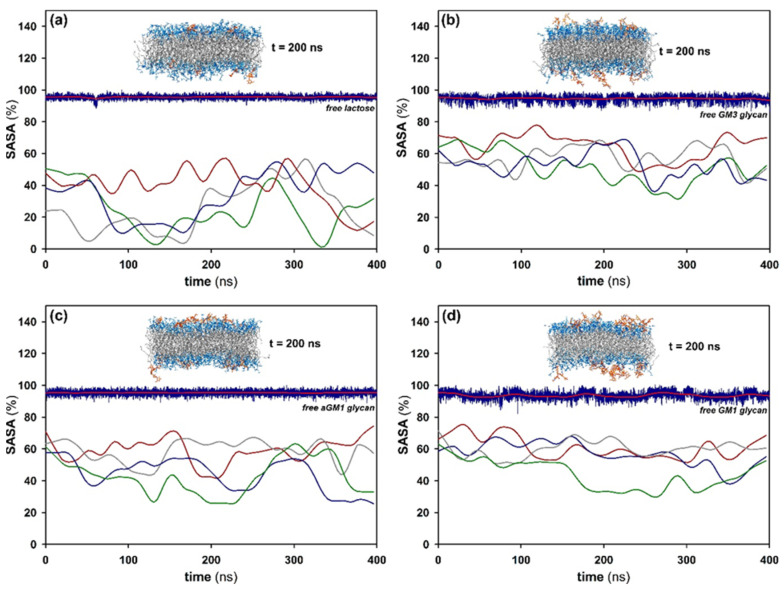
Solvent accessible surface area (SASA, %) along the simulation time referenced to free glycan moiety (straight line around 100% in all panels): (**a**) POPC:LacCer bilayer (system 2), reference: lactose; (**b**) POPC:GM3 bilayer (system 3), reference: 3′SL; (**c**) POPC:aGM1 bilayer (system 4), reference: aGM1 tetrasaccharide; (**d**) POPC:GM1 bilayer (system 5), reference: GM1 pentasaccharide. Raw and smoothed SASA data are shown for each free glycan. The behavior of four randomly chosen sugar moieties in each case are followed during the time course of the simulation (graphs in the bottom part of the panels). Snapshots at 200 ns are displayed for each bilayer model at the upper part of the panel. Color code: glycan residues in orange; phosphocholine (PC) in blue; palmitoyl (PA), oleoyl (OL), and ceramide (CER) moieties in grey.

A visual inspection of the glycan’s behavior along the trajectories resulting from the simulations (Figure S5 in SI) highlighted the high flexibility of the glycan moieties when located on the bilayer surface. In order to further examine their dynamic behavior, solvent accessible surfaces areas (SASA, %) were plotted using the corresponding free glycan as a reference (Figure 7, straight red line and dark blue plot around 100% in all panels). In the analysis, SASA was calculated for the Galactose residue that binds at the galectin binding site, which for GM1 and aGM1 it was shown by STD to be the terminal one. Figure 7 shows the SASA computed along the MD simulations for systems 2–5. Each line represents one randomly chosen Gal residue among the 16 included in each system. The SASA of the monitored residue fluctuates periodically along the simulation time, being always smaller than that for the free glycan in solution, as expected, but being particularly smaller in the case of LacCer. SASA values show that Gal residues of LacCer are only exposed to the solvent around 30%, while being around 60% for systems 3, 4, and 5.

**Figure 8:**
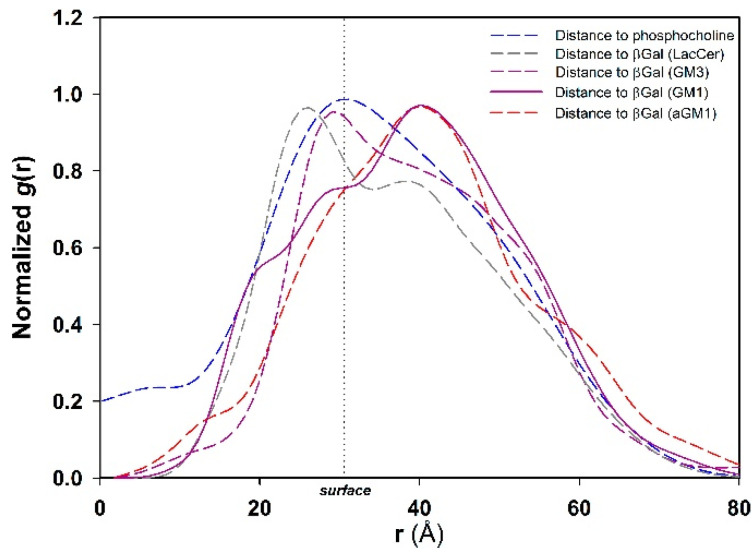
Radial distribution function, g(r), for all bilayers using g(r) to phosphocholine (PC) residue as reference. For bilayers 1, 3, and 4, g(r) was calculated from the center of mass of all lipid atoms to the terminal β-Gal residue, whereas non-terminal β-Gal residue was selected for bilayer 3.

Next, the distance between the key Gal residue (the terminal residue for GM1 and aGM1) and the lipid bilayer was monitored for each system, expressed as a radial distribution function, g(r) of the distance from the center of mass of all lipid atoms (equivalent to the geometric center of the lipid bilayer) to the geometric center of the key Gal residue. As a reference, the g(r) to the geometric center of the phosphocholine moiety was employed (Figure 7, blue dashed line). It is evident that the terminal Gal residue of aGM1 and GM1 is further away from the membrane surface, with a maximum population at g(r) ca. 40 Å, than that of the other GSLs. In fact, GM3 and LacCer display maxima at g(r) ca. 25 Å, that is at the surface level or even slightly hidden, while showing a shoulder at higher values. The combination of the SASA with the g(r) values (Figure 8) provide a hint for the experimental observation of a better interaction of the GM1 and aGM1-based liposomes with the galectins. The glycan moieties are accessible, and the population of exposed Gal moieties is large to provide interaction points with the lectins. The Gal moiety of GM3 and LacCer are much less exposed, from the solvent accessible perspective and are also closer to the membrane surface.

**Figure 9:**
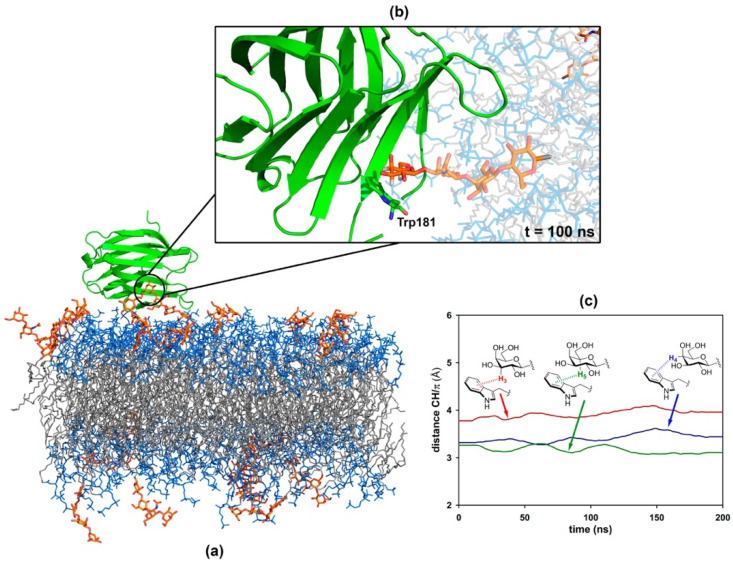
The CH-π interaction between the terminal β-Gal residue in aGM1 and Trp181 of Gal3 CRD is kept along the MD simulation. (**a**) Simulation of the interaction of the POPC:aGM1 bilayer with Gal3; (**b**) Snapshot (t = 100 ns) showing the key CH/π interaction with the key β−Gal residue of aGM1; (**c**) Distances (Å) between Gal H-3, H-4 and H-5 and the centroid of Trp181 aminoacid. Color code: Gal3, in green; glycan residues in orange; phosphocholine (PC) in blue; palmitoyl (PA), oleoyl (OL) and ceramide (CER) moieties in grey.

A 3D perspective of the interaction was then provided. Atomistic MD simulations (200 ns) of the interaction of bilayer 3 (aGM1) with hGal-3 were carried out (Figure 9). The molecular recognition event displays all the key features of the interaction of hGal3 with βGal moieties, as described [46]. It is noteworthy to verify the essential CH/π interaction between Trp181 and Gal H-3, H-4 and H-5 with a tripod-type spatial orientation [47]. F Indeed, the presentation of the glycans account for the permanent CH/π interaction (Figure 9) during all the simulation time.

## 3. Discussion

The NMR-based experimental data demonstrates a different tendency in the interaction ability of the glycosphingolipids with the galectins than the corresponding oligosaccharides. In free solution, 3′SL provides much better interactions than any other glycan, due to the additional interaction provided by the sialic acid moiety to key amino acid residues located at the S5 strand. This is especially notorious for the 3′SL/Gal8N interaction that is in the slow exchange regime in the chemical shift timescale (Figure S2D for Supplementary Material). Although the Neu5Ac residue is also present in GM1 and aGM1 oligosaccharides, NMR data clearly indicates that it does not participate in direct binding contacts with the galectins. While in a solution, the Gal and the sialic acid residues of GM3 are well exposed to provide the key intermolecular contacts, this is not the case in the liposome’s environment. Herein, only the relatively long GM1 and aGM1 gangliosides display the proper accessibility of the terminal Gal moiety to provide the impetus for the interaction (Scheme 1). Within the liposome, the Gal residue in LacCer and GM3 is not permanently exposed to let the interaction to take place in an efficient manner. MD simulations support this view of the role of the presentation of the glycan to establish the essential binding contacts.

Besides the presentation of the glycans, the obtained data also suggests that different partners (galectins in this case) may provide distinct interactions with the membrane. Herein, perturbations have been observed on the membrane surface of GM3, GM1, and aGM1 containing liposomes only upon the addition of Gal8N, and not of Gal3 (Figure 7), even though both lectins mostly recognize the same epitopes on the GSLs.

Moreover, it has been proposed, using coarse-grained and atomistic simulations, that in membranes containing GM3 and GM1, clusters are formed that differ in size, with GM3 clusters being larger and more stable than those of GM1 [48]. Moreover, only in the atomistic simulations, it was reported that the terminal monosaccharide moieties of the headgroups, Neu5Ac for GM3 and βGal for GM1 contribute to the cis-interactions to generate the clusters.

The different results obtained using different experimental approaches to tackle molecular recognition events point out the tremendous difficulty of translating in vitro results to the in vivo environment. Care should be taken when extracting conclusions from experiments conducted under specific conditions. Molecular recognition details may differ from solution to surfaces to membrane-like environments. The presentation of the interacting sugar epitope is essential and, therefore, the length and chemical nature of the linkers used to attach the ligands to surfaces should influence the obtained results. In our particular context, galectins are ubiquitous proteins, and their biological activity has been reported to take place at the cell surface, but they are also involved in intracellular trafficking and around disrupted vesicles, among other locations [49]. In most of these events, galectins may be in close contact to membranes that contain their saccharide partners. The in vivo scenario is even more complex since we must consider that all cells have a glycocalix that is formed not only with glycolipids but also heavily glycosilated extracellular proteins. Thus, depending on the presentation in the membrane or in solution, the binding to the preferred epitopes may subtly vary, highlighting the remarkable complexity of these phenomena in vivo and the difficulty of understanding these interaction processes using reductionistic approaches.

## 4. Materials and Methods

### 4.1. Materials

Glycans: 3′-Sialyllactose, asialo-GM1, and GM1 saccharide were purchased from Carbosynth.

Lipids: eggPC (l-α-phosphatidylcholine 840051), gangliosides (GM3 860058 and GM1 860065), and lactosylceramide (d-lactosyl-β-1,1′-*N*-stearoyl-d-erythro-sphingosine 860598) were purchased from Avanti Polar Lipids. Asialoganglioside GM1 (G3018) was obtained from Sigma-Aldrich (St. Louis, MO, USA).

Fluorescent probe 6-dodecanoyl-2-dimethylaminonaphthalene (Laurdan) from Invitrogen (Waltham, MA, USA).

All other materials (salts and organic solvents) were of analytical grade.

### 4.2. Protein Expression and Purification

The protein expression and purification are described elsewhere [50,51]. Briefly, proteins’ sequence in pET11a vector was transformed in BL21(DE3) *E. coli*. For labelled protein, a 5 mL overnight culture was added in one liter of M9 media containing antibiotic and ^15^N-NH_4_Cl (1 g) as the nitrogen source. When the culture reached 0.7–1.2 of OD_600_, protein expression was induced by the addition of 1 mM Isopropyl β-D-1-thio-galactopyranoside (IPTG) and growth continued for 3 h at 37 °C. After, cells were harvested and resuspended in a column buffer (PBS 1× pH 7.2, 2 mM EDTA, 2 mM β-mercarptoethanol /DTT, 0.1% NaN3) and 1 mM PMSF was added to inhibit proteases cleavage. The suspension was sonicated and the crude extract clarified by centrifugation at 35,000× *g* rpm for 30 min at 4 °C. The soluble fraction was loaded onto a pre-equilibrated α-Lactose-Agarose resin (Sigma-Aldrich) column and washed with 50 mL of column buffer. To elute recombinant h-Gal8N-ter 7 mL of elution buffer (150 mM α-Lactose in column buffer) was injected. Protein purity was checked by SDS-PAGE and subsequently confirmed by LC-MS.

Both galectins were thoroughly dialyzed against PBS, pH 7.4 until no lactose was present, before use.

### 4.3. Liposome Preparation

Powdered lipids were dissolved in an organic solution (2:1 chloroform: methanol, *v*/*v*). The desired amount was transferred to a glass vial, evaporates to dryness under a N2 stream, and kept under a vacuum for 2 h to remove all organic solvents. The obtained lipid film was then hydrated with the desired buffer (PBS pH 7.4) and vigorously vortexed to obtain multilamellar vesicles. After, the sample was subjected to 10 freeze and thaw cycles and extruded through 0.1 μm pore-size Nucleopore filters using a mini-extruder. Large Unilamellar Vesicles (LUVs) of 100 nm diameters were obtained.

The lipid concentrations were varied depending on the assay performed. The liposomes were prepared in at a final lipid concentration of 30 mM, being 28:2 mM PC:glycolipid when 10 equivalents of glycolipids were included, with respect to the lectin and 29.8:0.2 mM when one equivalent of glycolipids were included with respect to the lectin.

### 4.4. NMR Experiments

All spectra were performed at 298 K, on Bruker AVANCE 2 600 MHz and 800 MHz Bruker spectrometers equipped with cryoprobes.

The ^1^H NMR resonances of the ligands (3′-Sialyllactose, asialoGM1, and GM1 saccharides) were assigned through TOCSY (60 and 90 ms mixing times), NOESY (500–600 ms mixing times), and HSQC experiments using the Bruker AVANCE 2 600 MHz spectrometer equipped with standard triple-channel probe (600 MHz). Ligands were dissolved in deuterated phosphate buffered saline solutions at a concentration of 1 mM.

For saturation-transfer difference (STD NMR) experiments 30–50 μM of full- length galectin 3 were prepared in deuterated PBS and 70 equivalents of ligand was added. The on-resonance frequency was set at the aliphatic region (~0.77 ppm) and the off-resonance frequency at −25 ppm. To achieve protein saturation, a series of 25–50 ms PC9 pulses was used with a total saturation time of the protein of 2 s in the 600 MHz spectrometer. A spin-lock filter (100 ms) was used to remove the NMR signals of the macromolecule.

To analyze the binding of sugar moiety of gangliosides to galectins and calculate affinities ^1^H−^15^N-HSQC were recorded on 50 μM ^15^N-labeled hGal-3 CRD, at 298 K. Chemical shift perturbations (CSP) were followed and dissociation constants (KD) were calculated using the CcpNmr Analysis 2.4.2 software. ^1^H−^15^N-HSQC were recorded analyzing the binding of galectins to liposomes. After, the peak volumes of the cross-peaks were measured, using the CcpNmr Analysis 2.4.2 software [52].

### 4.5. Membrane Polarization

Liposomes were prepared as described above, including a 0.5% Laurdan in DMSO. Samples emission spectra (400 nm to 600 nm) were acquired in a Quanta Master 40 spectrofluorometer (Photon Technology International, Birmingham, AL, USA) setting the wavelength excitation at 360 nm. GP values were estimated with the equation GP = ((I_440_ − I_490_)/(I_440_ + I_490_)), where I_440_ and I_490_ corresponded to the emission intensity at 440 nm and 490 nm, respectively.

### 4.6. Model Building and MD Details

The initial geometries of the heterogeneous lipid bilayers were built using the CHARMM membrane generator [53]. In all cases, a rectangular box (X and Y initial length of 100 Å) was generated containing a 14:1 ratio of randomly distributed 1-palmitoyl-2-oleoyl-sn-glycero-3-phosphocholine (POPC, 140 lipids) and the corresponding glycosylceramide (10 glycolipids) in each leaflet. In addition, a water thickness of 17.5 Å (TIP3P) [54] above and below the membrane was also included. Next, some water molecules were replaced by ions (NaCl) up to a fixed concentration of 150 mM, following a Monte-Carlo (MC) approach [55].

In order to check that the POPC membrane was correctly generated, the area per lipid was calculated. The computational value of the area per lipid was 65.2 Å2, fairly close to the experimentally obtained previously [56] for the liquid crystalline phase of POPC bilayers (64.3 ± 1.3 Å^2^) at 30 °C.

To prepare the glycolipid unit, the crystallographic coordinates of ceramide (18:1/18:0) moiety were retrieved from the Protein data base (PDB ID: 5J14). This structure was optimized using Gaussian 16 package [57] to generate the “true” minimum (Nimag = 0). Next, RESP charges were generated at HF/6-31G(d) level to be consistent with AMBER and GLYCAM parametrization [58]. A two-stage fitting was employed assigning the same partial charge for the aliphatic protons (atomic charge equilibration predicted by atom paths). Similar to the lipid17 force field, these partial charges were not fixed to zero. An in-house script was applied to change the atom types to be consistent with the extension to lipids and glycolipids of GLYCAM_06 force field [59]. Systems containing Gal-3 (PDB ID: 1KJL) were prepared aligning the principal axis to z and then by translating the protein 45 Å along the z-axis.

System equilibration and MD simulations. The Amber20 software [55] with GLYCAM_06j-1, ff14SB [60] and lipid17 [61] force field parameters were applied for glycolipids, proteins, and lipids, respectively. Five different steepest descent/conjugate gradient minimizations (maximum 20,000 steps) were carried out on the initial structures of POPC:Glycolipid bilayers. In the first minimization, the positions of POPC, glycolipids, and ions were restrained using harmonic potential restraints with a force constant k of 100 kcal mol-1 Å-2. For the second minimization, POPC and glycolipids were only restrained. Subsequent minimizations were performed without any restraints. In these steps, temperature was maintained by using a Langevin thermostat [62]. After minimization, the system was gradually heated increasing the temperature in two steps: (a) from 10 to 100 K during 5 ps (NVT assemble) and (b) from 100 to 303 K at NPT assemble (100 ps) using a Berendsen barostat (1 bar) [63]. Assuming periodic boundary conditions (PBC), the particle mesh Ewald method was employed for modelling long-range electrostatic interactions, whereas the cutoff for non-bonded interactions was set to 10 Å. The SHAKE algorithm [64] was used to constraint covalent bonds involving hydrogen atoms. Next, a 5 ns simulation (10 steps, 500 ps each step) in the NPT assemble was performed to equilibrate the system. The final snapshot of this equilibration was used as a jumping-off point for MD production under the same conditions. Four consecutive MD simulations (100 ns each) was run to obtain the final production trajectory. Three independent replicas were carried out for each system. To check the soundness of the generated models within the simulation time, potential energy, and RMSD values were employed. In all cases, the analysis of the RMSD showed that these POPC–glycolipid membranes reached a geometric equilibrium after 15 ns (Figure S4 in Supplementary Materials). In addition, these systems were completely stable, displaying potential energy values of −2.19, −3.01, −2.58, and −2.55 105 kcal/mol for 1–4, respectively.

Data analysis. Solvent Accessible Surface Area (SASA) was estimated to assess the availability of the glycan epitope to interact with the lectin in the liposome environment. SASA was calculated along the simulation time by using the cpptraj module. Raw data were referenced to the maximum value (in Å2) obtained for the free ligand in a water box (100% SASA). Next, a bisquared smoothing algorithm with a sampling proportion of 0.1 was applied for the final plot representation (Figure 7).

## Figures and Tables

**Table 1:** Dissociation constant values (K_D_) (µM) for the interaction of Gal3 and Gal8N with lactose, 3′SL, and GM1 pentasaccharide and aGM1 tetrasaccharide estimated from ^1^H,^15^N-HSQC titrations. Data from the literature are also included.

Glycan	Gal3-CRD			Gal8-N		
	NMR	(a)	(b)	NMR	(b)	(c)
Lactose	125 ± 13	2.8	260	108 ± 15	130	3.1/1.7
3′SL	70 ± 8	1.7	230	- ^i^	0.6	0.05
GM1 Pentasaccharide	180 ± 27		130	152 ± 26	30	4.1
aGM1 Tetrasaccharide	307 ± 35		180	82 ± 10	46	6.7

(**a**) from Ref [27]; (**b**) from Ref [30]; (**c**) from Ref [42]. ^i^ In slow exchange. Low micromolar. Not quantitatively determined.

## Data Availability

Not applicable.

## Supplementary Materials

The following are available online at https://www.mdpi.com/article/10.3390/ph15020145/s1, Figure S1. 1H-STD-NMR spectra of the interaction of Gal8N. Figure S2. 1H-15N HSCQ-NMR spectra showing the CSP of Gal3 upon addition of increasing concentration of the saccharide moiety. Figure S3. Surface charge of Gal3 and Gal8N. Figure S4. RMSD plots. Figure S5. Initial (t = 0 ns) and final (t = 400 ns) snapshots. Figure S6. 1H assignment of asialo-GM1 and GM1.
