# Supplementary material for: Oligosaccharide Presentation Modulates the Molecular Recognition of Glycolipids by Galectins on Membrane Surfaces
Source: Pharmaceuticals (Basel). 2022 Jan 26;15(2):145. doi: 10.3390/ph15020145 (PMC8878398; doi:10.3390/ph15020145)
Supplement: Supplementary file 1 [file pharmaceuticals-15-00145-s001.zip › pharmaceuticals-1515169-supplementary.pdf]

Supporting

Figure S1. ^{1}H-STD-NMR spectra of the interaction of Gal8N

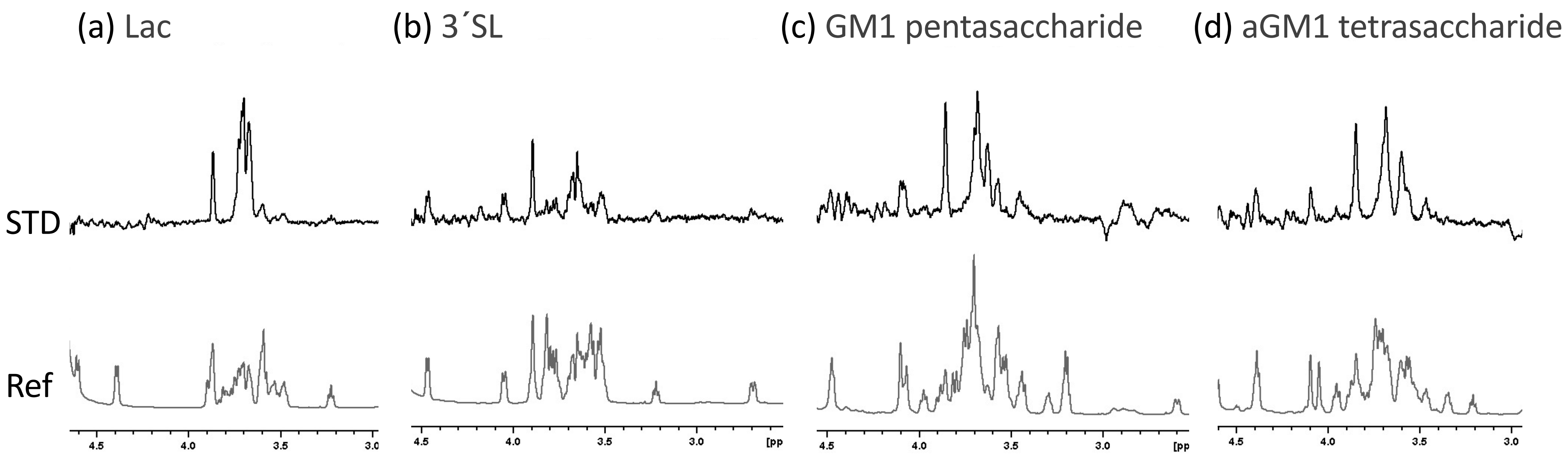

Figure S2. ^{1}H-^{15}N HSCQ-NMR spectra showing the CSP of Gal3 (50 μM) upon addition of increasing concentration of the saccharide moiety of (A) GM3 (B) asialo-GM1(C) GM1, and of Gal8N (50 μM) (D) GM3 (E) asialo-GM1(F) GM1. All titrations were done with at least 7different ligand concentrations. In this panel 4 different titration points are shown: blue protein alone, red 1 eq, yellow 5 eq, and green 10 eq of ligand. Except for panel D: blue protein alone, red 0.5 eq, yellow 1 eq, and green 1.5 eq of ligand.

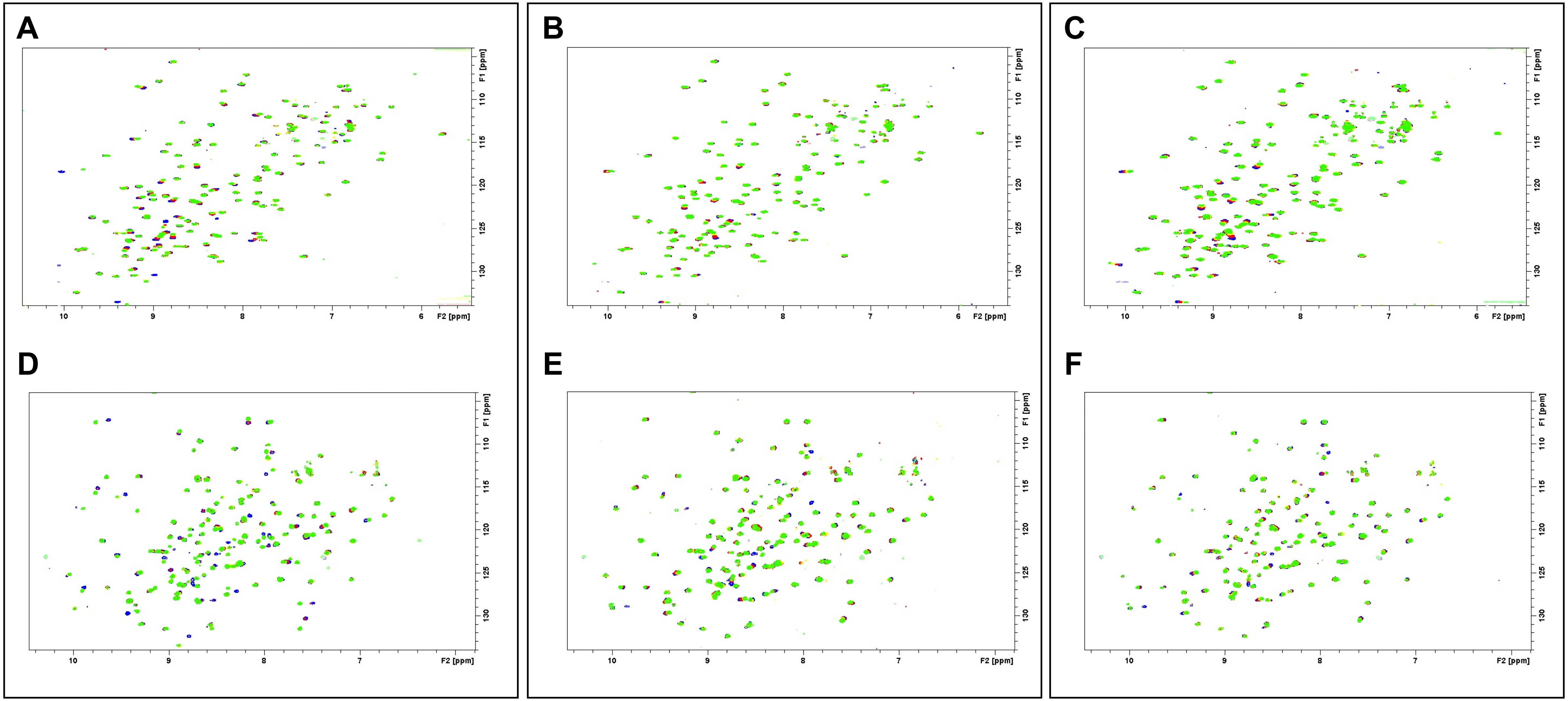

Figure S3. Surface charge of Gal3 and Gal8N

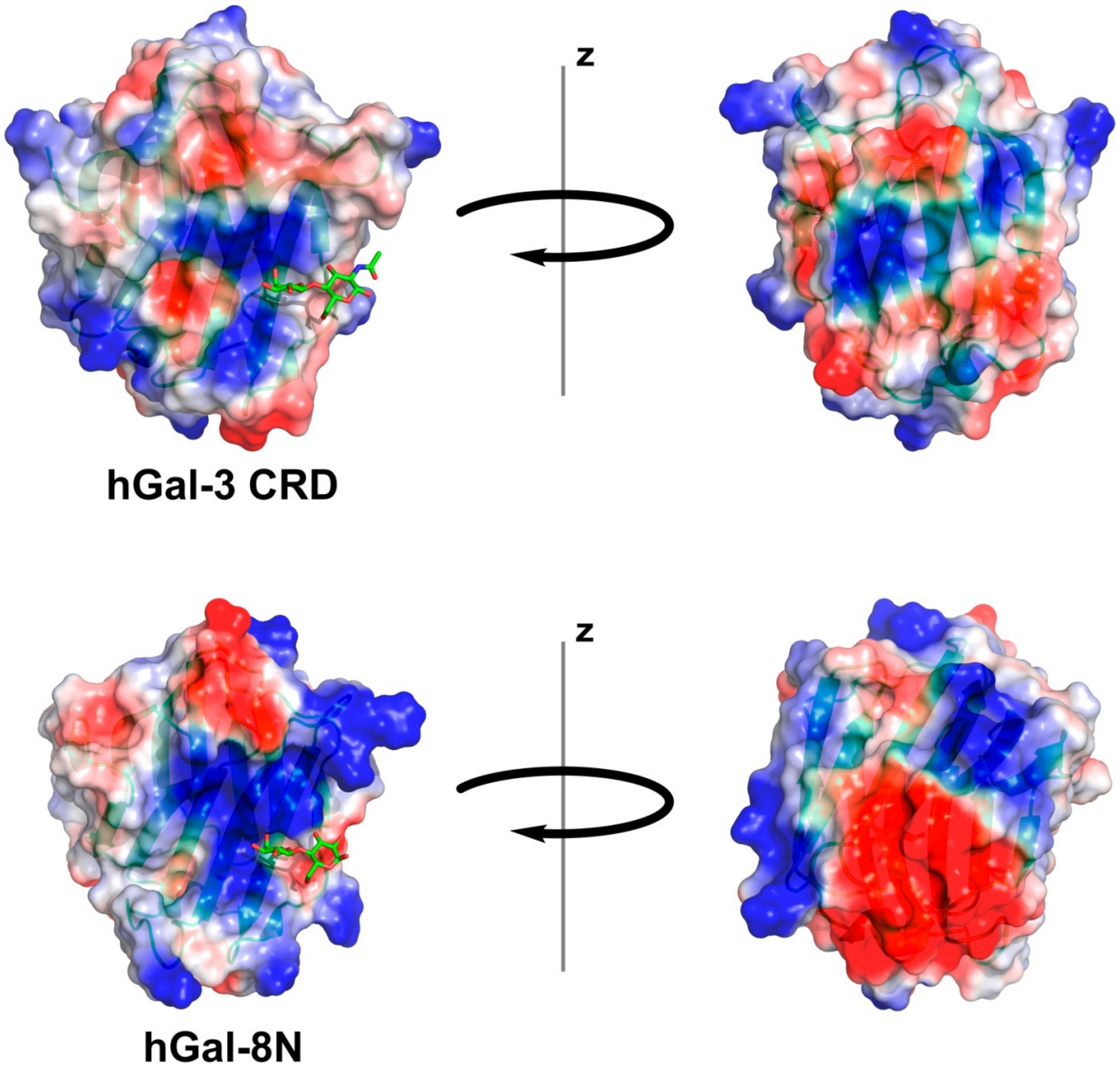

Figure S4. RMSD plots for (**A**) POPC:LacCer bilayer; (**B**) POPC:GM3; (**C**) POPC:aGM1; (**D**) POPC:GM1 and (**E**) Recognition of POPC:aGM1 bilayer by hGal3.

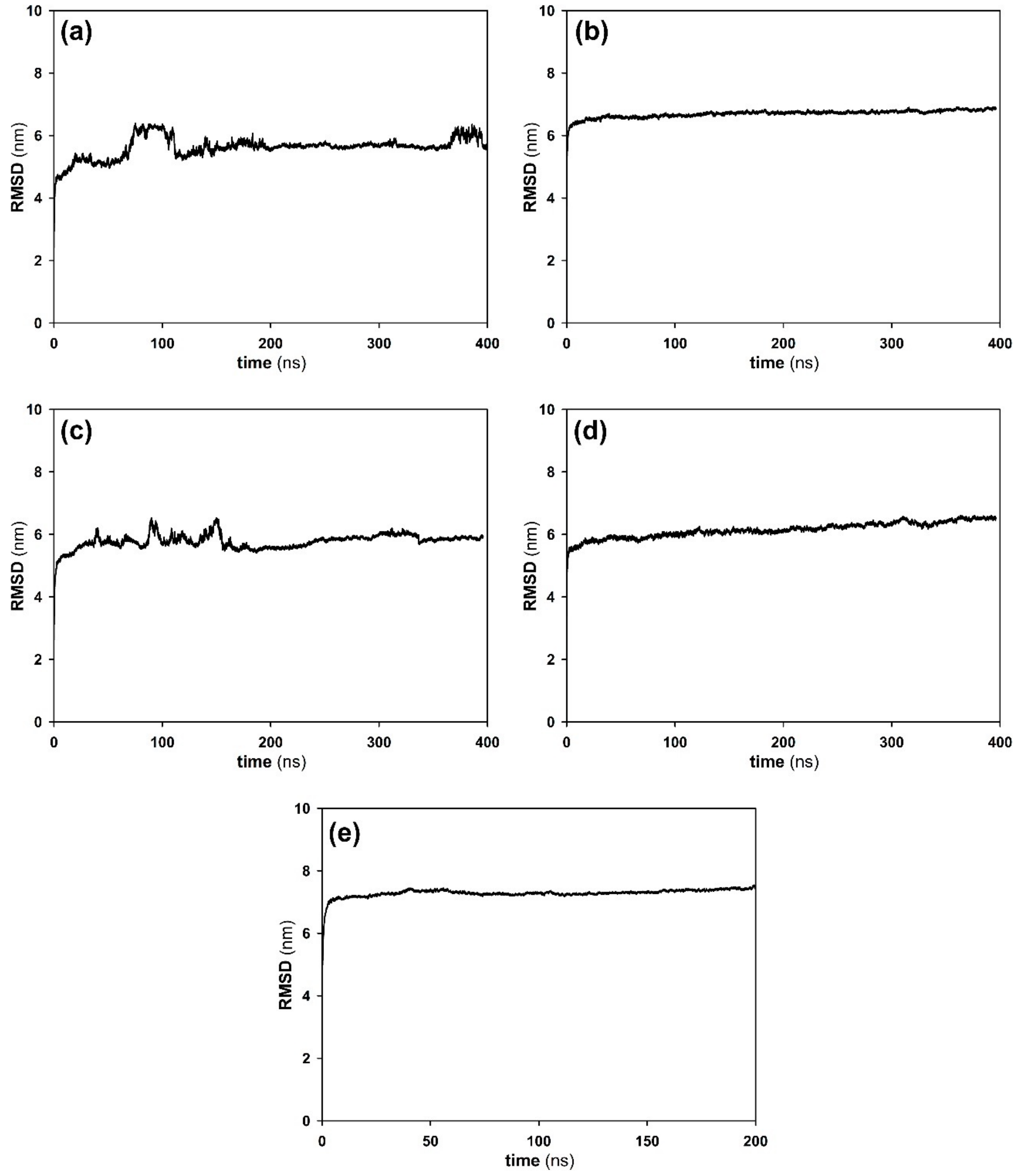

Figure S5. Initial (t = 0 ns) and final (t = 400 ns) snapshots of (**A**) POPC:LacCer bilayer; (**B**) POPC:GM3 bilayer; (**C**) POPC:aGM1 and (**D**) POPC:GM1 bilayer.

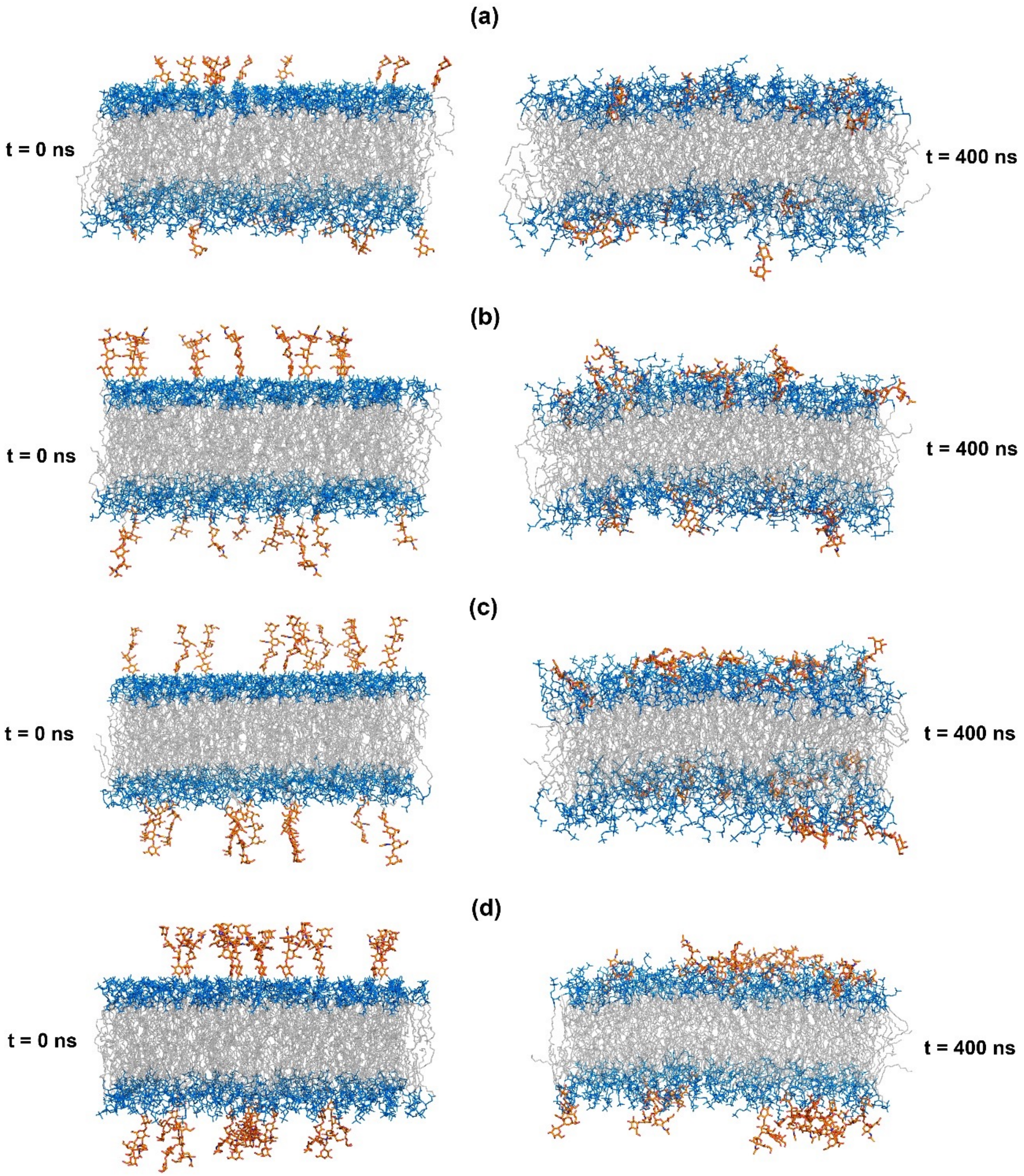

Figure S6. ^{1}H assignment of asialo-GM1 (A) and GM1 (B) of internal galactose residue (Gal_int) and of external galactose (Gal_ex) residues.

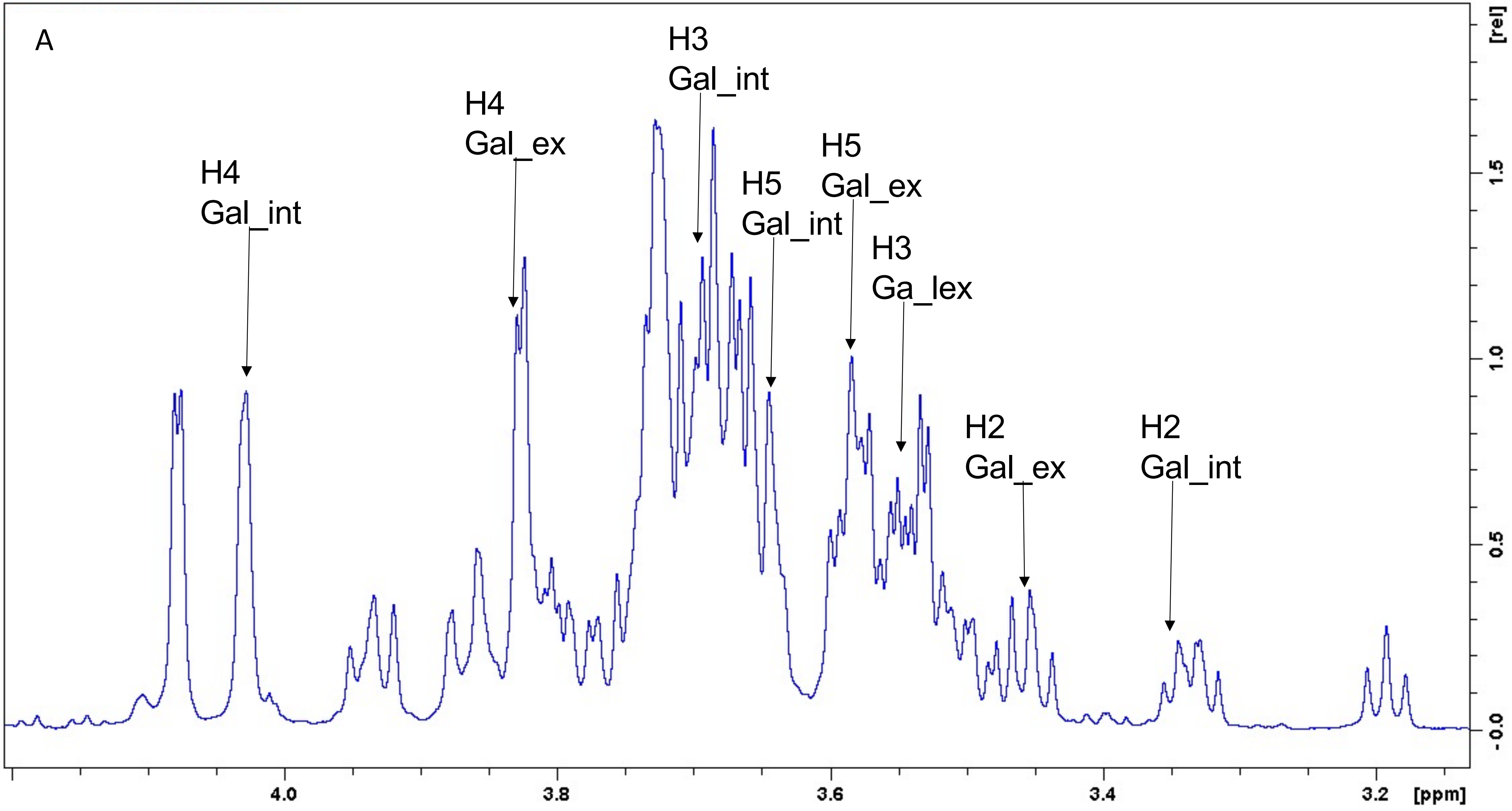

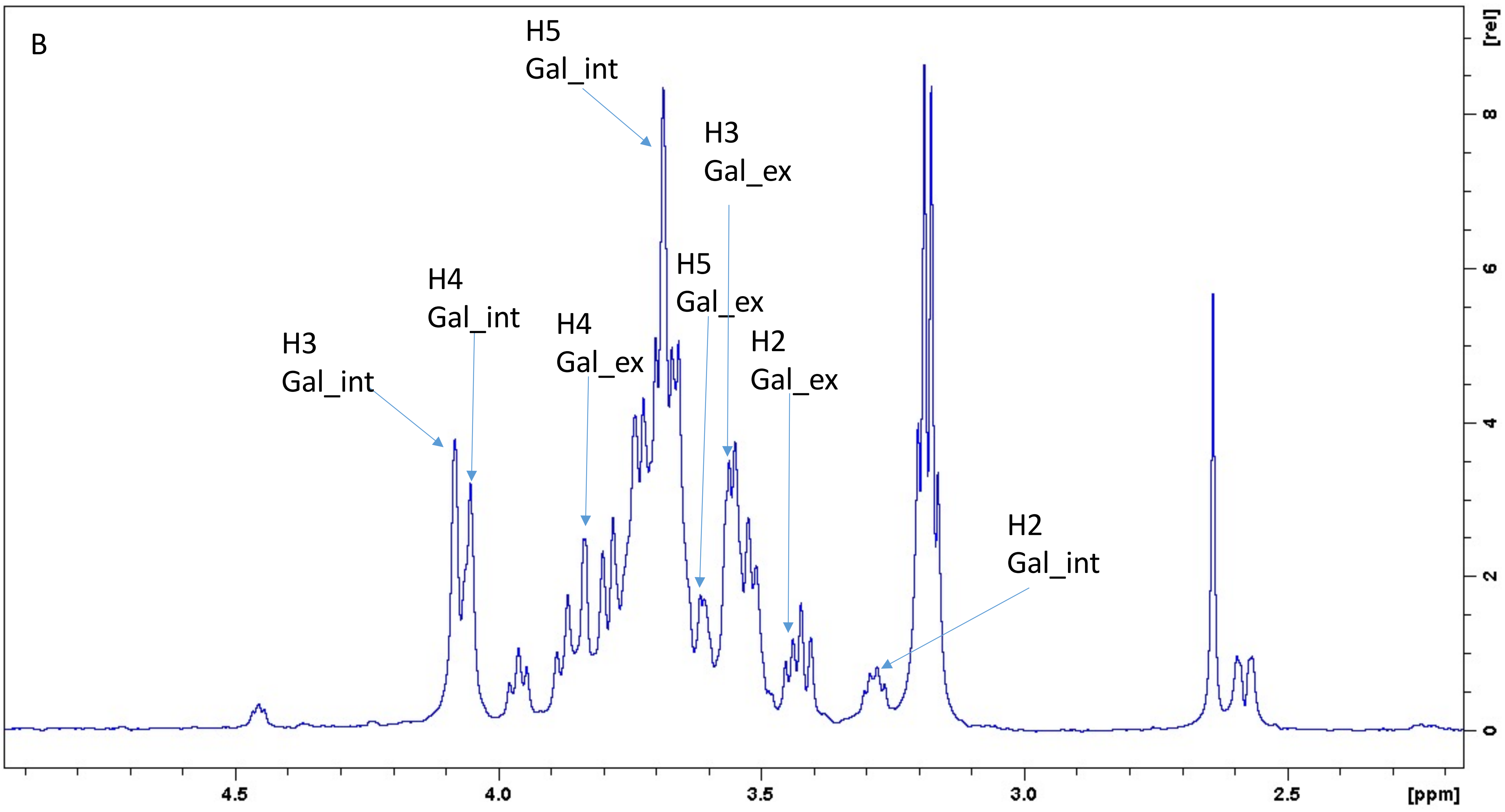
B
H5
Gal_int
H3
Gal_ex
H5
Gal_ex
H4
Gal_int
H4
Gal_ex
H2
Gal_ex
H3
Gal_int
H2
Gal_int
4.5
4.0
3.5
3.0
2.5
[ppm]
[rel]
8
6
4
2
0
